# Quality of life among family caregivers of cancer patients: an investigation of SF-36 domains

**DOI:** 10.1186/s40359-023-01399-6

**Published:** 2023-12-19

**Authors:** Mina Rostami, Mahsa Abbasi, Morteza Soleimani, Zhaleh Karimi Moghaddam, Alireza Zeraatchi

**Affiliations:** 1 Social Determinants of Health Research Center, Zanjan University of Medical Sciences, Zanjan, Iran; 2 Psychology Center, Ayatollah Mousavi Hospital, Zanjan University of Medical Sciences, Zanjan, Iran; 3 Master of Clinical Psychology, Department of Clinical Psychology, Islamic Azad University Science and Research Unit, Tehran, Iran; 4 Department of Radiation Oncology, School of Medicine, Vali-e-Asr Hospital, Zanjan University of Medical Sciences, Zanjan, Iran; 5 Department of Emergency Medicine, School of Medicine, Ayatollah Mousavi Hospital, Vali-e-Asr Hospital, Zanjan University of Medical Sciences, Zanjan, Iran

**Keywords:** Quality of life, Family caregivers, Cancer patient

## Abstract

**Background:**

With improving survival rates, cancer has become more of a chronic disease with long-term palliative care requirements. Thus, it is even more than ever necessary to pay careful attention to the well-being of family caregivers of cancer patients, as cancer trajectory is a challenging path for both patients and their caregivers. This study focusses on ascertaining the level of quality of life (QoL) domains and their attributable significant factors among a population of cancer family caregivers.

**Methods:**

This was a cross-sectional study. The study population consist of caregivers of adult cancer patients in Zanjan, Iran between 2019 and 2020. Medical Outcomes General Health Survey Short Form 36 (SF-36) was the instrument to measure outcome variables. Clinical and basic characteristics of the caregivers and their patients were also collected using a questionnaire designed for this purpose. Data were analyzed using Independent samples t-test, Analysis of Variance, and stepwise linear regression in SPSS v.26.

**Results:**

Of the caregivers 167 were male and 133 were female. The mean age of the participants was 40.77 ± 12.56, most of whom were offspring of the patients (148, 49.3%), married (239, 79.7%), and self-employed (81, 27.0%). both domains of bodily pain (76.50 ± 16.67) and physical functioning (74.88 ± 20.27) showed the highest scores among caregivers. Age and gender of caregivers, duration of caregiving, Eastern Cooperative Oncology Group (ECOG) performance status scale as well as type and stage of cancer, and type of treatment were among the significant predictors of QoL domains (All, p < 0.001).

**Conclusion:**

Findings of the present study substantiated various significant predictors for QoL along with low levels of QoL domains among the caregivers of cancer patients. Securing such findings proves the magnitude of probable unmet needs and psychological challenges in this population and provides the health policy makers with some valuable clues to draw effective strategies to address such issues.

## Background

Thanks to the increased survival and decreased mortality in the population of cancer patients in recent decades in developed countries [1], the course of the disease has altered greatly to become more of a chronic disease with long-term palliative care requirements. Thus, caregiving for cancer patients has become of enormous importance, being considered one of the necessities in the cancer trajectory [2].

Dealing with cancer impacts the quality of life (QoL) for family caregivers. It has been well shown that cancer doesn’t solely affect patients; it also has a significant impact on various aspects of their families’ well-being, particularly their psychological well-being [3]. Assuming the role of a caregiver for a cancer patient presents distinct difficulties. Caregivers must offer both emotional and practical support to the patient while also managing their own emotions tied to the diagnosis, all in an effort to maintain resilience for the well-being of the patient [4]. This is especially challenging given the demanding and stressful nature of the disease and treatment, which affects both the patient and their family caregivers. Thus, it appears essential to investigate the quality of life (QoL) of family caregivers of cancer patients and the factors that play a role in it as a fundamental aspect of cancer care and management.

Sociodemographic factors such as age, gender, education status, occupation, and income, as well as cancer clinical characteristics, and different treatment strategies have previously been demonstrated to be some of the predicting factors for QoL among this population [3, 5]. Regarding the duration of caregiving, the positive correlation between care time and caregivers’ QoL has previously been reported in a population of Iranian caregivers of breast cancer patients [6].

Caregivers’ QoL is strongly influenced by factors such as the patient’s condition, the caregiver’s gender, the amount of time spent on caregiving, and more importantly, their psychological state [7]. Lack of sufficient social support, poor mental health and caring for patients with lower functional status were significant influencing factors of lower QoL in caregivers of adults with cancer [8].

In a population of caregivers of patients with multiple myeloma, lower QoL was linked to poor financial status [9]. Age, gender, educational level, occupation, economic status, average duration of caregiving, and age of the patient were significantly associated with the level of quality of life in an Iranian population of caregivers of children with cancer [10]. Lower levels of education have been found to be significantly related to a poorer QoL in the physical, social, and environmental domains. Both employed and married caregivers have been shown to have significantly greater likelihood of reporting a better QoL in psychological domain [11]. Moreover, caregiving for patients with advanced stage cancers along with being a male caregiver have been suggested to be significant predictors of low QoL [12].

Studying such factors allows health policy-makers and clinicians to examine and monitor the quality of life and unmet needs of this population to implement effective health-promoting and preventive measures when needed [5].

Being a subjective value, QoL is not a permanent concept and alters over time. It is a dynamic, multicomponent concept, encompassing a spectrum of individual’s life aspects as well as their needs, beliefs, values and attitudes. In fact, the individual is in a constant effort to strike a balance between the real situation and the ideal situation [13].

Yet, there has been numerous tools to measure QoL, one of which is SF-36. Being known as an acknowledged tool for the appraisal of QoL level, SF-36 has been used to assess QoL among medically non-ill population and cancer patients [14]. Functional status and well-being that are approved concepts within the “health” definition, have been considered as conceptual framework for SF-36 evolution, so that the QoL measured using this instrument is health-related quality of life [15].

While there is a wealth of research on the quality of life experienced by caregivers of cancer patients, our study intends to address a gap in the existing literature by delving into the specific sub-scales of the SF-36 QoL questionnaire and the factors influencing them. Therefore, we aimed to achieve three imperative objectives by conducting this study, (1) The level of the QoL domains among caregivers of cancer patients (2) The significant influencing factors of QoL domains through a univariate analysis (3) The potential predictors of QoL domains using a multivariable regression analysis.

## Methods and materials

### Study design and subjects

Using a cross-sectional design, a population of family caregivers of adult cancer patients were studied at Vali-e-Asr Hospital in Zanjan, Iran during 2019–2020. The study focused on individuals who served as primary family caregivers for cancer patients and were selected based on eligibility criteria using a convenience sampling approach. The caregivers were associated with patients who had medical records and were currently undergoing treatment under the oversight of the principal investigator. A face to face interview approach was utilized, based on an individual collection method. A trained interviewer used plain and easily comprehensible language to orally present the questionnaire to the illiterate participants. Considering caregivers of inpatients, the interview was conducted upon their discharge. For outpatients, the interview carried out during their clinic follow-up visit. All interviews were conducted in a private and quiet room within the hospital, which was chosen to ensure the privacy and comfort of the participants.

### Ethical considerations

The present study was evaluated and approved by the Ethics Committee of Zanjan University of Medical Sciences [IR.ZUMS.REC.1398.105]. Before starting the interview, participants were informed about the study’s purpose, the confidentiality of their responses, and their rights as participants. A written informed consent was obtained from all participants. In terms of illiterate participants the informed consent was obtained from their legal guardian(s).

### Eligibility criteria

The inclusion criteria encompassed individuals aged ≥ 18 years, being unpaid and informal family caregivers who and had been taking the role of caregiving for at least 6 months with a major contribution in caring for the patient. We excluded participants with a history of psychological or disabling physical conditions in addition to those who were unable to respond to the questionnaire properly.

### Measurements

Eight domains of quality of life consisted the outcome variables which were measured by trained researchers using Persian-version of SF-36 questionnaire. Variables of gender, age, education, marital status, relationship to the patient under care and duration of patient care were also included as effect modifiers. Patient related data such as gender, stage of cancer, time since cancer diagnosis, care setting (Inpatient or outpatient), type of treatment (Radiation therapy or combination of radiation and chemotherapy), and (ECOG) performance status were considered as confounding variables.

### SF-36 questionnaire

It is a 36-item self-reporting questionnaire to assess QoL by measuring mental and physical health through eight sub-scales including, physical functioning (10 items), role physical (4 items), bodily pain (2 items), general health (5 items), vitality (4 items), social functioning (2 items), role emotional (3 items) and mental health (5 items). Total score of each sub-scale ranges from 0 to 100 with higher scores showing a greater QoL. A review of studies revealed that majority of studies has reported a Cronbach’s α coefficient for all 8 SF-36 sub-scales ≥ 0.70 [16].

In Iran, Asghari Moghaddam and Faqihi have evaluated the reliability and validity of Persian-version of SF-36 among both clinical and non-clinical subjects. Cronbach’s α coefficient for all subjects ranged from 0.70 for role physical and role emotional to 0.85 for physical functioning. With regard to test-retest reliability, coefficients have been reduced to some extent, so that the lowest coefficient has been reported for role emotional (0.43) and the highest for general health (0.79). The validity of questionnaire has been reported excellent, differentiating clinical and non-clinical subjects in all 8 sub-scales [17].

### Statistical analysis

Shapiro-Wilk’s test and Box-Plot were used to test the normality of data distribution. Normally-distributed numerical data were represented using mean ± standard deviation (SD) and non-normal numerical data were reported as median (InterQuartile Range [IQR]). Categorical data were shown as frequency (%). To draw a comparison between two groups based on the outcome variables, we used independent samples t-test. Analysis of Variance (ANOVA) was carried out to compare ≥ 3 groups with Tukey Honestly Significant Difference (HSD) post-hoc test if equal variances assumed. Otherwise, Welch’s ANOVA was used as an alternative with Games-Howell post-hoc test. Stepwise linear regression analysis was done using dummy coded variables to procure possible predicting factors for QoL domains. A p-value less than 0.05 (two-sided) was considered statistically significant. All data were analyzed using SPSS software version 26 (SPSS Inc., Chicago, IL, USA).

## Results

### Basic characteristic of the participants

Of 300 family caregivers, 167 (55.7%) were male and 133 (44.3%) were female. Mean ± SD age of the caregivers was 40.77 ± 12.56. Most of them were offspring of the patients (148, 49.3%), married (239, 79.7%), and self-employed (81, 27.0%) (Table 1).

**Table 1:** Basic and clinical characteristics of the patients and caregivers

Patients	Mean ± SD / N (%)	Caregivers	Mean ± SD / N (%)
**Age, years**	52.94 ± 14.33	**Age, years**	40.77 ± 12.56
**Gender, female**	164 (54.7)	**Gender, male**	167 (55.7)
**Type of cancer**		**Marital status**	
Breast	39 (13.0)	Married	239 (79.7)
Prostate	34 (11.3)	Single	61 (20.3)
Bladder	22 (7.3)	**Education**	
Stomach	61 (20.3)	Illiterate	44 (14.7)
Esophagus	30 (10.0)	Primary school	44 (14.7)
Colorectal	41 (13.7)	Junior high school	37 (12.3)
Brain	18 (6.0)	Senior high school	11 (3.7)
Lung	55 (18.3)	HSD	77 (25.7)
**Stage of cancer**		Associate degree	27 (9.0)
1	30 (10.0)	BS	37 (12.3)
2	55 (18.3)	MSc and above	23 (7.7)
3	94 (31.3)	**Relationship to patient**	
4	121 (40.3)	Spouse	72 (24.0)
**ECOG**		Offspring	148 (49.3)
0	149 (49.7)	Parents	38 (12.7)
1	91 (30.3)	Siblings	19 (6.3)
2	42 (14.0)	Others	23 (7.7)
3	12 (4.0)	**Employment status**	
4	6 (2.0)	Governmental employed	61 (20.3)
**Type of treatment**		Self-employed	81 (27.0)
Chemo + Radiation therapy	74 (24.7)	retired	53 (17.7)
Radiation therapy	22 (7.3)	unemployed	80 (26.7)
Surgery	31 (10.3)	Quit for care	25 (8.3)
Chemotherapy	151 (50.3)	**Family income**	
Radio + Hormone therapy	15 (5.0)	≤ 40,000,000 IRR / Month	87 (29.0)
Chemo + Hormone therapy	7 (5.0)	40,000,000–80,000,000 IRR / Month	99 (33.0)
**Time since diagnosis, month**		≥ 80,000,000 IRR / Month	114 (38.0)
6–11	170 (56.7)	**Presence of other caregivers**	
12–23	61 (20.3)	Yes	62 (20.8)
≥ 24	69 (23.0)	No	238 (79.3)
**Care setting**		**Duration of caregiving, month**	
Inpatient	152 (50.7)	6–11	183 (61.0)
Outpatient	148 (49.3)	12–23	57 (19.0)
**Health insurance**		≥ 24	60 (20.0)
Public health insurance	118 (39.3)		
Social security insurance	98 (32.7)		
Armed forces medical services insurance	19 (6.3)		
supplemental insurance	65 (21.7)		

Of 300 patients, the majority were female (164, 54.7%). The average age of the patients was 52.94 ± 14.33. Stomach (61, 20.3%), lung (55, 18.3%) and colorectal (41, 13.7%) cancers were the most common cancers. Chemotherapy (151, 50.3%) and Chemo + Radiation therapy (74, 24.7%) were the most frequent treatment strategies. The most common type of insurance was public health insurance (118, 39.3%). (Table 1).

**Fig. 1:**
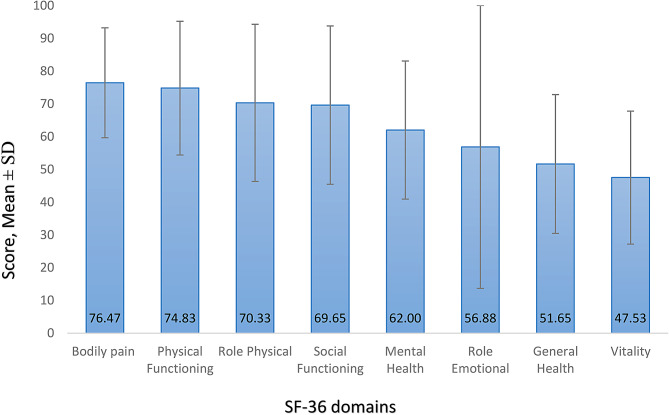
SF-36 domains among family caregivers. Columns represent means and error bars represent Standard Deviations (SD)

In terms of SF-36 domains both domains of bodily pain (76.50 ± 16.67) and physical functioning (74.88 ± 20.27) showed the highest scores among caregivers. (Fig. 1; Table 2)

**Table 2:** Mean and standard deviation of SF-36 domains

SF-36 domains	Mean	SD	Min	Max
Physical Functioning	74.83	20.43	0	100
Role Physical	70.33	23.99	0	100
Bodily Pain	76.47	16.78	10	100
General Health	51.65	21.17	5	95
Vitality	47.53	20.30	5	90
Social Functioning	69.65	24.18	0	100
Role Emotional	56.88	43.20	0	100
Mental Health	62.00	21.08	10	90

### Univariate analysis

#### Physical functioning

Mean physical functioning differed significantly between different conditions of caregivers’ age groups (Welch’s ANOVA, P = 0.012), so that caregivers ≤ 30 had significantly better physical functioning compared to those ≥ 61 years (mean, 80.23 vs. 67.50, P = 0.010) and 51–60 years (mean, 80.23 vs. 68.28, P = 0.048).

Relationship to the patients also showed a significant effect (F(4, 295) = 2.804, P = 0.026). Post hoc analysis revealed that being a spouse was related to significantly lower levels of physical functioning compared to those who had not an immediate relation (mean, 69.72 vs. 84.35, P = 0.023).

There was a significant association between employment status and Physical Functioning (Welch’s ANOVA, P = 0.001). Caregivers who were governmental employed had significantly better physical functioning compared to retired (mean, 81.64 vs. 64.25, P < 0.0001) and unemployed (mean, 81.64 vs. 77.31, P = 0.002).

ECOG had a significant effect on the score of bodily pain (F(4, 295) = 4.170, P = 0.003). Caregiving for patients with ECOG 4 (53.33 ± 11.25) caused significantly worse levels of Bodily Pain compared to ECOG 0 (77.58 ± 19.56, P = 0.032).

No significant associations were found between other basic characteristic of the participants and Physical Functioning (All, P > 0.05). (Tables 3 and 4)

**Table 3:** Comparison of quality of life scales in terms of basic characteristics of the family caregivers

Caregivers	Physical Functioning		Role Physical		Bodily Pain		General Health		Vitality		Social Functioning		Role Emotional		Mental Health	
	Mean ± SD	p-value	Mean ± SD	p-value	Mean ± SD	p-value	Mean ± SD	p-value	Mean ± SD	p-value	Mean ± SD	p-value	Mean ± SD	p-value	Mean ± SD	p-value
**Gender**																
Female	77.03 ± 19.33	0.097†	70.86 ± 24.84	0.733†	75.49 ± 16.15	0.367†	51.32 ± 20.44	0.808†	46.65 ± 20.30	0.504†	69.44 ± 24.69	0.892†	57.13 ± 42.56	0.928†	61.84 ± 21.72	0.908†
Male	73.08 ± 21.16		69.91 ± 23.37		77.25 ± 17.27		51.92 ± 21.79		48.23 ± 20.34		69.82 ± 23.84		56.67 ± 43.83		62.13 ± 20.62	
**Age, year**																
≤ 30	80.23 ± 15.69	**0.012‡**	71.15 ± 20.83	0.800*	77.97 ± 14.02	0.695‡	53.15 ± 21.48	0.726*	63.38 ± 13.81	< 0.0001*	71.69 ± 22.55	0.841*	67.16 ± 37.96	0.099‡	63.23 ± 21.96	0.488*
31–40	75.78 ± 19.51		71.67 ± 24.05		76.67 ± 16.32		53.17 ± 22.46		47.78 ± 19.63		69.50 ± 24.49		55.54 ± 44.41		60.78 ± 21.41	
41–50	76.49 ± 19.16		67.16 ± 25.07		77.75 ± 13.41		51.27 ± 20.80		45.37 ± 17.88		69.78 ± 23.39		59.19 ± 43.35		64.18 ± 19.39	
51–60	68.28 ± 22.23		71.88 ± 28.22		72.41 ± 23.60		49.22 ± 22.29		44.38 ± 19.90		65.47 ± 24.14		47.90 ± 43.12		64.38 ± 22.71	
≥ 61	67.50 ± 25.66		70.11 ± 23.93		74.93 ± 19.91		48.80 ± 18.08		30.00 ± 16.53		69.78 ± 27.42		47.82 ± 45.89		57.83 ± 20.48	
**Marital status**																
Married	74.64 ± 20.65	0.752†	70.92 ± 24.39	0.402†	76.59 ± 16.88	0.800†	52.53 ± 21.14	0.154†	45.44 ± 19.73	**< 0.0001†**	69.69 ± 24.64	0.959†	54.10 ± 43.86	**0.027†**	61.63 ± 20.85	0.550†
Single	75.57 ± 19.72		68.03 ± 22.42		75.98 ± 16.51		48.20 ± 21.09		55.74 ± 20.59		69.51 ± 22.46		67.74 ± 38.96		63.44 ± 22.07	
**Education**																
Illiterate	67.16 ± 26.00	0.198**‡**	68.75 ± 28.59	0.644*	68.75 ± 28.59	0.424*	47.27 ± 19.77	0.675*	36.70 ± 21.04	**0.002‡**	70.23 ± 25.72	0.759*	50.74 ± 43.42	**0.024‡**	56.48 ± 23.36	0.259*
Primary school	71.70 ± 21.99		72.73 ± 23.38		76.48 ± 17.24		51.14 ± 20.90		42.50 ± 17.40		66.82 ± 27.02		38.62 ± 40.63		66.36 ± 19.02	
Junior high school	78.51 ± 19.39		72.97 ± 21.55		77.08 ± 17.52		54.86 ± 20.01		49.59 ± 19.09		71.62 ± 26.30		51.34 ± 46.87		67.57 ± 19.84	
Senior high school	80.00 ± 21.79		77.27 ± 28.40		79.27 ± 14.19		54.09 ± 22.00		47.27 ± 5.64		59.55 ± 20.30		57.57 ± 49.64		57.73 ± 18.07	
HSD	75.78 ± 15.49		69.16 ± 21.80		77.68 ± 16.04		53.31 ± 23.09		53.90 ± 21.33		72.21 ± 20.17		64.92 ± 41.15		60.84 ± 19.65	
Associate degree	81.48 ± 17.47		71.30 ± 23.72		77.04 ± 13.25		47.41 ± 18.93		51.85 ± 20.62		68.15 ± 24.30		69.12 ± 39.15		60.37 ± 24.57	
BS	74.32 ± 20.07		64.19 ± 26.70		80.05 ± 13.11		51.35 ± 21.81		50.81 ± 19.84		67.70 ± 25.78		66.65 ± 40.07		60.81 ± 21.42	
≥MS	76.96 ± 22.19		73.91 ± 20.61		72.26 ± 16.56		54.57 ± 21.31		43.04 ± 17.94		71.96 ± 24.57		55.06 ± 45.62		65.00 ± 22.20	
**Relationship to patient**																
Spouse	69.72 ± 23.25	0.026*	70.14 ± 25.04	0.752*	74.04 ± 16.65	0.055*	50.28 ± 23.36	0.454*	41.74 ± 19.89	0.008*	67.64 ± 27.80	0.620*	31.47 ± 38.30	< 0.0001*	60.49 ± 23.52	0.826*
Offspring	76.45 ± 19.65		71.79 ± 23.01		75.04 ± 17.40		53.89 ± 20.71		49.73 ± 20.95		71.32 ± 22.02		72.06 ± 38.11		61.72 ± 20.41	
Parents	74.08 ± 16.59		67.11 ± 22.59		81.34 ± 13.9		47.63 ± 19.30		50.66 ± 19.03		67.11 ± 23.55		28.93 ± 37.30		65.39 ± 20.54	
Siblings	71.58 ± 22.30		65.79 ± 25.29		81.21 ± 13.63		49.21 ± 18.87		39.21 ± 17.81		73.68 ± 25.64		75.43 ± 42.80		61.32 ± 18.24	
Others	84.35 ± 16.53		70.65 ± 28.85		81.30 ± 17.68		50.22 ± 21.71		53.26 ± 16.48		66.09 ± 25.84		69.55 ± 40.10		63.48 ± 21.39	
**Employment status**																
Governmental employed	81.64 ± 18.63	**0.001‡**	69.26 ± 24.74	0.468*	76.34 ± 15.58	0.290‡	47.95 ± 21.84	0.056*	49.51 ± 19.65	< 0.0001*	72.13 ± 22.95	0.381‡	56.82 ± 43.18		64.18 ± 22.89	0.650*
Self-employed	75.37 ± 19.16		67.90 ± 23.79		78.01 ± 17.19		57.16 ± 21.03		48.46 ± 18.61		66.05 ± 24.01		57.60 ± 42.82	0.216*	59.75 ± 20.20	
Retired	64.25 ± 25.21		68.40 ± 24.09		73.36 ± 20.70		47.74 ± 19.10		34.43 ± 20.13		67.45 ± 27.38		45.90 ± 45.86		61.60 ± 20.63	
Unemployed	77.31 ± 17.53		73.44 ± 23.64		78.25 ± 15.28		51.50 ± 20.08		55.19 ± 18.46		71.63 ± 24.00		64.15 ± 41.35		63.69 ± 21.16	
Quit for care	71.00 ± 17.85		75.00 ± 23.93		72.68 ± 12.79		51.60 ± 25.07		43.00 ± 20.20		73.60 ± 20.59		54.65 ± 42.90		59.40 ± 20.58	
**Family income**																
≤ 40,000,000 IRR / Month	73.28 ± 21.65	0.700	73.85 ± 22.52	0.231*	77.14 ± 14.97	0.192*	50.57 ± 21.15	0.846*	44.83 ± 19.13	0.244‡	65.29 ± 24.67	0.119*	55.16 ± 45.13	0.464*	59.02 ± 21.57	0.232*
40,000,000–80,000,000 IRR / Month	75.35 ± 19.81		67.93 ± 23.70		78.37 ± 18.39		51.87 ± 21.14		47.78 ± 22.71		70.51 ± 23.29		61.27 ± 41.71		64.29 ± 20.34	
≥ 80,000,000 IRR / Month	75.57 ± 20.13		69.74 ± 25.21		74.31 ± 16.52		52.28 ± 21.37		49.39 ± 18.87		72.24 ± 24.31		54.37 ± 43.06		62.28 ± 21.24	
**Presence of other caregivers**																
Yes	75.16 ± 20.04	0.887†	69.35 ± 25.37	0.719†	73.19 ± 15.70	0.084†	48.95 ± 20.70	0.261†	50.08 ± 21.75	0.268†	68.95 ± 24.31	0.799†	54.82 ± 41.86	0.675†	63.87 ± 19.88	0.434†
No	74.75 ± 20.58		70.59 ± 23.67		77.32 ± 16.97		52.35 ± 21.28		46.87 ± 19.90		69.83 ± 24.19		57.41 ± 43.61		61.51 ± 21.39	
**Duration of caregiving, month**																
6–11	77.08 ± 18.34	0.057 ‡	70.36 ± 24.42	0.998*	77.20 ± 17.14	0.500*	58.77 ± 18.89	**< 0.0001‡**	49.64 ± 19.94	0.005*	70.36 ± 24.17	0.808*	60.46 ± 43.20	0.115*	69.21 ± 18.15	< 0.0001*
12–23	73.42 ± 22.28		70.18 ± 23.35		76.47 ± 15.65		50.61 ± 18.97		48.77 ± 19.46		68.95 ± 23.67		55.54 ± 42.88		58.16 ± 18.33	
≥ 24	69.33 ± 23.64		70.42 ± 23.69		74.25 ± 16.78		30.92 ± 15.30		39.92 ± 20.71		68.17 ± 24.99		47.21 ± 42.65		43.67 ± 19.97	

*P < 0.05, obtained from ANOVA F-test

† P < 0.05, obtained from independent samples t-test

‡ P < 0.05, obtained from Welch’s ANOVA

Significant P-values are shown in bold

HSD, High School Diploma; BS, Bachelor’s Degree, MS, Master’s Degree, SD, Standard Deviation

**Table 4:** Comparison of quality of life scales in terms of basic and clinical characteristics of the patients

Patients	Physical Functioning		Role Physical		Bodily Pain		General Health		Vitality		Social Functioning		Role Emotional		Mental Health	
	Mean ± SD	p-value	Mean ± SD	p-value	Mean ± SD	p-value	Mean ± SD	p-value	Mean ± SD	p-value	Mean ± SD	p-value	Mean ± SD	p-value	Mean ± SD	p-value
**Gender**																
Female	75.88 ± 19.58	0.329†	69.67 ± 24.23	0.663†	74.52 ± 17.67	**0.027†**	50.55 ± 21.04	0.323†	46.62 ± 18.84	0.391†	68.05 ± 24.64	0.208†	59.34 ± 41.95	0.280†	60.55 ± 21.53	0.191†
Male	73.57 ± 21.43		70.88 ± 23.86		78.82 ± 15.37		52.98 ± 21.33		48.64 ± 21.96		71.58 ± 23.56		53.91 ± 44.64		63.75 ± 20.46	
**Age, year**																
≤ 30	72.14 ± 23.52	0.367‡	70.00 ± 21.69	0.952*	76.74 ± 18.90	0.861*	50.29 ± 22.81	0.395*	43.29 ± 21.86	0.385*	74.86 ± 21.47	0.147*	45.70 ± 46.50	0.292*	59.29 ± 24.10	0.850*
31–40	77.08 ± 21.36		67.36 ± 28.54		74.56 ± 14.75		49.31 ± 19.20		49.86 ± 17.42		69.03 ± 25.99		56.47 ± 45.65		61.94 ± 22.49	
41–50	73.98 ± 22.66		70.76 ± 21.35		75.07 ± 17.09		56.44 ± 21.41		50.93 ± 20.66		73.64 ± 22.94		51.40 ± 42.58		64.41 ± 20.86	
51–60	71.56 ± 20.82		71.31 ± 23.64		77.21 ± 15.28		51.72 ± 21.83		47.79 ± 20.25		63.85 ± 25.71		61.19 ± 42.68		61.72 ± 19.34	
≥ 61	77.25 ± 17.35		70.64 ± 24.96		77.36 ± 17.50		50.23 ± 20.73		46.15 ± 20.48		69.27 ± 23.83		61.15 ± 41.70		61.74 ± 20.88	
**Care setting**																
Inpatient	75.95 ± 19.16	0.337‡	68.75 ± 24.62	0.248†	76.04 ± 17.01	0.653†	50.66 ± 21.36	0.412†	44.21 ± 18.42	**0.004†**	63.72 ± 26.31	**< 0.0001†**	47.57 ± 42.82	< 0.0001†	60.00 ± 20.81	0.096†
Outpatient	73.68 ± 21.66		71.96 ± 23.31		76.91 ± 16.59		52.67 ± 20.99		50.95 ± 21.61		75.74 ± 20.10		66.43 ± 41.60		64.05 ± 21.22	
**Type of cancer**																
Breast	74.49 ± 18.45	0.483*	75.64 ± 16.70	0.203‡	74.82 ± 19.70	0.709*	48.46 ± 20.33	0.837*	47.82 ± 21.48	0.350‡	72.82 ± 25.69	**0.014‡**	65.80 ± 42.91	0.017*	62.05 ± 20.82	0.427*
Prostate	73.38 ± 22.31		63.24 ± 21.52		76.09 ± 17.39		52.94 ± 21.57		58.24 ± 26.93		78.82 ± 22.89		68.62 ± 41.80		69.26 ± 19.03	
Bladder	69.77 ± 19.78		63.64 ± 21.44		75.23 ± 14.44		49.77 ± 23.11		44.32 ± 18.72		57.27 ± 24.04		31.80 ± 40.46		57.73 ± 21.42	
Stomach	77.54 ± 19.82		72.13 ± 27.04		79.00 ± 16.05		49.02 ± 22.11		46.56 ± 18.87		72.13 ± 24.63		64.47 ± 41.67		62.21 ± 18.65	
Esophagus	76.50 ± 20.47		72.50 ± 23.98		74.77 ± 16.52		53.83 ± 23.33		48.00 ± 17.79		60.67 ± 25.75		46.65 ± 40.68		64.50 ± 21.94	
Colorectal	75.24 ± 18.97		70.12 ± 25.75		74.12 ± 18.90		54.51 ± 20.39		44.76 ± 21.32		64.88 ± 26.65		49.58 ± 42.90		58.90 ± 23.78	
Brain	66.11 ± 25.64		72.22 ± 22.50		81.50 ± 16.79		53.61 ± 19.00		42.78 ± 16.46		76.94 ± 21.90		49.99 ± 47.48		57.78 ± 25.16	
Lung	76.64 ± 20.75		70.00 ± 26.08		76.60 ± 14.47		52.82 ± 20.31		46.45 ± 17.28		70.00 ± 17.61		58.17 ± 43.14		61.27 ± 20.84	
**Stage of cancer**																
1	75.17 ± 17.78	0.065	70.00 ± 22.16	0.442*	80.03 ± 15.50	0.619*	53.83 ± 19.01	0.845*	59.50 ± 21.78	0.007*	75.00 ± 21.29	0.001*	86.66 ± 31.07	< 0.0001*	69.50 ± 21.70	0.196*
2	73.27 ± 22.07		74.55 ± 22.81		77.15 ± 15.74		50.27 ± 20.62		46.73 ± 21.39		73.55 ± 22.45		68.47 ± 40.28		62.73 ± 22.66	
3	79.31 ± 18.35		70.74 ± 24.76		75.89 ± 17.70		52.55 ± 22.47		45.11 ± 19.48		74.36 ± 22.95		42.89 ± 40.79		61.28 ± 20.49	
4	71.98 ± 21.42		68.18 ± 24.36		75.73 ± 16.87		51.03 ± 21.06		46.82 ± 19.27		62.89 ± 25.18		55.08 ± 44.24		60.37 ± 20.48	
**ECOG Performance Status**																
0	77.58 ± 19.56	0.003*	72.65 ± 25.05	0.171*	78.40 ± 16.16	0.001*	52.58 ± 21.86	0.280*	45.44 ± 18.60	0.253*	72.99 ± 25.23	< 0.0001*	51.89 ± 42.16	0.279*	61.11 ± 21.29	0.532*
1	75.71 ± 22.04		68.96 ± 23.67		77.87 ± 16.93		53.41 ± 20.94		47.69 ± 20.52		70.60 ± 21.51		62.63 ± 42.99		60.77 ± 21.39	
2	69.40 ± 19.54		64.29 ± 22.86		72.26 ± 17.00		45.71 ± 19.36		51.79 ± 22.84		65.36 ± 20.55		63.48 ± 45.86		65.48 ± 20.29	
3	63.75 ± 11.89		66.67 ± 16.28		67.42 ± 14.22		46.25 ± 20.24		54.17 ± 24.57		49.17 ± 20.09		58.32 ± 45.23		69.17 ± 20.54	
4	53.33 ± 11.25		83.33 ± 12.91		55.00 ± 8.89		54.17 ± 18.28		54.17 ± 28.00		43.33 ± 33.11		44.43 ± 45.54		64.17 ± 18.28	
**Time since diagnosis, month**																
6–11	75.97 ± 18.32	0.136‡	69.56 ± 24.54	0.708*	77.09 ± 17.19	0.643*	58.97 ± 18.89	< 0.0001*	49.18 ± 19.91	0.002*	70.29 ± 24.04	0.864*	60.77 ± 43.26	0.158*	69.09 ± 18.27	< 0.0001*
12–23	77.13 ± 21.30		72.54 ± 22.22		76.57 ± 16.75		52.79 ± 19.11		51.23 ± 19.69		68.52 ± 24.90		54.63 ± 42.62		62.13 ± 18.67	
≥ 24	70.00 ± 23.87		70.29 ± 24.35		74.84 ± 15.87		32.61 ± 16.07		40.22 ± 20.30		69.06 ± 24.16		49.26 ± 43.01		44.42 ± 19.43	
**Type of treatment**																
Chemo + Radiation therapy	72.84 ± 21.47	0.602*	68.58 ± 25.19	0.366*	76.91 ± 18.09	0.740*	52.64 ± 20.71	0.339*	47.43 ± 19.14	0.102‡	68.24 ± 23.53	0.048*	45.03 ± 42.89	**< 0.0001‡**	58.58 ± 21.35	0.120*
Radiation therapy	78.41 ± 20.25		62.50 ± 26.44		75.55 ± 13.98		60.23 ± 19.96		51.36 ± 26.91		79.09 ± 18.42		46.96 ± 46.75		67.73 ± 20.68	
Surgery	76.77 ± 18.37		70.16 ± 22.74		74.94 ± 17.55		53.06 ± 18.87		55.32 ± 20.53		74.52 ± 22.99		83.86 ± 30.88		67.58 ± 22.39	
Chemotherapy	75.23 ± 19.73		73.01 ± 23.89		76.32 ± 16.35		49.87 ± 21.79		44.60 ± 18.77		66.52 ± 25.15		56.50 ± 42.86		61.52 ± 20.90	
Radio + Hormone therapy	68.67 ± 27.28		65.00 ± 20.70		82.40 ± 12.95		47.67 ± 21.20		56.67 ± 23.88		80.33 ± 20.04		73.32 ± 40.24		68.33 ± 17.28	
Chemo + Hormone therapy	80.71 ± 18.80		67.86 ± 12.19		72.00 ± 24.89		55.00 ± 24.49		45.71 ± 22.62		77.86 ± 26.90		66.65 ± 43.04		52.14 ± 18.22	
**Health insurance**																
Public health insurance	77.46 ± 18.27	0.145*	74.15 ± 22.39	0.090*	78.31 ± 15.08	0.406‡	52.42 ± 22.98	0.934*	53.01 ± 20.00	< 0.0001*	69.49 ± 22.95	0.272*	59.87 ± 42.60	0.534*	63.52 ± 22.27	0.658*
Social security insurance	71.38 ± 22.13		67.86 ± 24.34		75.84 ± 19.32		50.61 ± 20.81		40.61 ± 19.14		66.99 ± 25.35		52.03 ± 44.40		61.28 ± 19.48	
Armed forces medical services insurance	78.42 ± 21.01		61.84 ± 25.50		73.26 ± 18.17		52.63 ± 21.75		49.21 ± 21.93		78.16 ± 25.06		63.14 ± 41.42		63.95 ± 18.60	
supplemental insurance	74.23 ± 20.93		69.62 ± 25.19		75.03 ± 15.14		51.54 ± 18.34		47.54 ± 19.36		71.46 ± 24.12		56.91 ± 43.19		59.77 ± 22.03	

*P < 0.05, obtained from ANOVA F-test

† P < 0.05, obtained from independent samples t-test

‡ P < 0.05, obtained from Welch’s ANOVA

Significant P-values are shown in bold

HSD, High School Diploma; BS, Bachelor’s Degree, MS, Master’s Degree, SD, Standard Deviation

#### Role physical

No significant associations were found between basic characteristic of the participants and Role Physical (All, P ≥ 0.05). (Tables 3 and 4)

#### Bodily Pain

ECOG had a significant effect on the score of bodily pain (F(4, 295) = 4.877, P = 0.001). Caregiving for a patient with ECOG 4 (55.00 ± 8.89) caused significantly lower scores of Bodily Pain compared to ECOG 0 (78.40 ± 16.16, P = 0.006) and ECOG 1 (77.87 ± 16.93, P = 0.009).

Caregiving for male patients was associated with significantly higher scores of bodily pain compared with caregiving for female patients (mean, 78.82 vs. 74.52, t(298) = -2.219, P = 0.027).

No significant links were found between other basic characteristic of the participants and Bodily Pain (All, P > 0.05). (Tables 3 and 4)

#### General Health

Duration of caregiving demonstrated a significant effect on General Health (Welch’s ANOVA, P < 0.0001). Caregiving for patients ≥ 24 months (30.92 ± 15.30) was associated with significantly lower levels of General Health compared with 6–12 (58.77 ± 18.89, P < 0.0001) and 12–23 (50.61 ± 18.97, P < 0.0001) months. The difference between 12 and 23 and 6–11 months was also significant (P = 0.015).

Likewise, time since diagnosis had a significant effect on General Health (F(2, 297) = 50.873, P < 0.0001), so that, caregivers showed significantly lower levels of general health if they had been caregiving for patients ≥ 24 months (32.61 ± 16.07) since diagnosis, compared to 12–23 (52.79 ± 19.11, P < 0.0001) and 6–11 (58.97 ± 18.89, P < 0.0001) months.

No significant associations were seen between other basic characteristic of the participants and Bodily Pain (All, P > 0.05). (Tables 3 and 4)

#### Vitality

Mean score of Vitality was significantly different between caregivers’ age groups (F(4, 295) = 24.881, P < 0.0001). Participants who were ≤ 30 years (63.38 ± 13.81) had significantly higher levels of Vitality in comparison with other age groups (All, P < 0.0001). Additionally, ≥ 61 group (30.00 ± 16.53) represented significantly lower levels of Vitality compared with 31–40 (47.78 ± 19.63, P < 0.0001), 41–50 (45.37 ± 17.88, P < 0.0001) and 51–60 (44.38 ± 19.90, P = 0.004) groups.

Single caregivers showed significantly more levels of Vitality compared to married ones, t(298) = 3.605, P < 0.0001.

There was a significant association between education and Vitality (Welch’s ANOVA, P = 0.002).

Games-Howell post-hoc test showed that caregivers with high school diploma (HSD) (53.90 ± 21.33) reported higher levels of Vitality compared with those who were illiterate (36.70 ± 21.04, P = 0.001) and at primary school (42.50 ± 17.40, P = 0.039) level.

Relationship to the patient had a significant effect on Vitality (F(4, 295) = 3.492, P = 0.008). The only pairwise significant relationship was seen between offspring and spouse, so that, offspring’ of patients reported significantly higher levels of Vitality compared to spouses (49.73 vs. 41.74, P = 0.045).

Employment showed a significant association with Vitality (F (4, 295) = 9.905, P < 0.0001). Retired caregivers (34.43 ± 20.13) reported significantly lower levels of Vitality in comparison with governmental employed (49.51 ± 19.65, P < 0.0001), self-employed (48.46 ± 18.61, P < 0.0001) and unemployed (55.19 ± 18.46, P < 0.0001) caregivers. Also, unemployed caregivers reported significantly higher levels of Vitality compared with quit for care (43.00 ± 20.20, P = 0.047).

There was a significant link between duration of caregiving and Vitality (F (2, 297) = 5.474, P = 0.005). Being a caregiver ≥ 24 (39.92 ± 20.71) months resulted in significantly lower levels of Vitality compared with 6–11 (49.64 ± 19.94, P = 0.003) and 12–23 (48.77 ± 19.46, P = 0.046) months.

Similarly, the relationship between time-since-diagnosis and Vitality was also significant (F (2, 297) = 6.256, P = 0.007). Caregivers showed significantly lower levels of Vitality if they had been caregiving for patients ≥ 24 months (40.22 ± 20.30), compared to 12–23 (51.23 ± 19.69, P = 0.005) and 6–12 (49.18 ± 19.91, P = 0.005) months.

Caregivers of outpatients demonstrated significantly greater levels of Vitality compared to inpatients (50.95 vs. 44.21, t(298) = 3.605, P = 0.004).

There was a significant association between stage of cancer and Vitality (F(3, 296) = 4.124, P = 0.007). Caregivers of patients with stage 1 (59.50 ± 21.78) cancer reported significantly higher levels of Vitality compared with stage 2 (46.73 ± 21.39, P = 0.027), stage 3 (45.11 ± 19.48, P = 0.004) and stage 4 (46.82 ± 19.27, P = 0.011).

Type of health insurance had a significant effect on the score of Vitality (F(3, 296) = 7.106, P < 0.0001). The only significant pairwise comparison was between public health (53.01 ± 20.00) and social security (40.61 ± 19.14) insurances, P < 0.0001.

No significant associations were seen between other basic characteristic of the participants and Vitality (All, P > 0.05). (Tables 3 and 4)

#### Social functioning

Caregivers of outpatients demonstrated significantly higher levels of Social Functioning compared to inpatients (75.74 vs. 63.72, t(298) = -4.439, P < 0.0001).

Type of cancer had a significant effect on score of Social Functioning (Welch’s ANOVA, P = 0.014). The only significant pairwise comparison was between prostate (78.82 ± 22.89) and bladder (57.27 ± 24.04) cancers, P = 0.034.

There was a significant link between stage of cancer and Social Functioning (F(3, 296) = 5.546, P = 0.001). Caregivers of patients with stage 4 (62.89 ± 25.18) cancer reported significantly lower levels of Social Functioning compared to stage 2 (73.55 ± 22.45, P = 0.030), and stage 3 (74.36 ± 22.95, P = 0.003) cancers.

Post-hoc analysis with Tukey-HSD revealed that caregiving for patients with ECOG 0 (72.99 ± 25.23) was associated with significantly higher scores of social functioning compared with ECOG 3 (49.17 ± 20.09, P = 0.007) and 4 (43.33 ± 33.11, P = 0.022). Similarly, ECOG 1 also was associated with better social functioning compared to ECOG 3 (P = 0.027) and 4 (P = 0.049).

There was a significant association between type of treatment and Social Functioning (F (5, 294) = 2.271, P = 0.048). Post-hoc test showed no significant pairwise comparisons (All, P ≥ 0.05).

No significant associations were observed between other basic characteristic of the participants and Social Functioning (All, P > 0.05). (Tables 3 and 4)

#### Role emotional

Caregivers of outpatients showed significantly higher levels of Role Emotional compared to inpatients (66.43 vs.47.57, t(298) = -3.867, P < 0.0001).

Married caregivers showed significantly lower levels of Role Emotional compared to single ones (54.10 vs. 67.74, t(298) = -2.216, P = 0.027).

Education had a significant effect on the score of Role Emotional (Welch’s ANOVA, P = 0.024). Having been graduated from a primary school (38.62 ± 40.63) was associated with significantly lower levels of Role Emotional compared to HSD (64.92 ± 41.15, P = 0.021), and B.S. (66.65 ± 40.07, P = 0.050).

The association between relationship-to-patient and Role Emotional was significant (F (4, 295) = 20.311, P < 0.0001). Caregivers showed significantly lower levels of Role Emotional if they were parents of patients (28.93 ± 37.30) compared to offspring’s (72.06 ± 38.11, P < 0.0001), siblings (75.43 ± 42.80, P = 0.003) and others (69.55 ± 40.10, P = 0.003). Being a spouse (31.47 ± 38.30) was also related to significantly lower levels of Role Emotional in comparison with offspring’s (P < 0.0001), siblings (P = 0.003) and others (P = 0.003).

There was a significant association between type of cancer (F (7, 292) = 2.487, P = 0.017) and Role Emotional, so that, caregiving for patients with bladder cancer (31.80 ± 40.46) was related to significantly lower levels of Role Emotional compared to prostate (68.62 ± 41.80, P = 0.036) and stomach (64.47 ± 41.67, P = 0.045) cancers.

There was a significant link between stage of cancer and Role Emotional (Welch’s ANOVA, P < 0.0001). Caregiving for patients with stage 3 (42.89 ± 40.79) cancer was associated with significantly lower scores of Role Emotional compared to stage 1 (86.66 ± 31.07, P < 0.0001) and 2 (68.47 ± 40.28, P = 0.002). Likewise, stage 4 was associated with significantly lower scores (55.08 ± 44.24) compared to stage 1 (P < 0.0001).

The relationship between type of treatment and Role Emotional was also significant (Welch’s ANOVA, P < 0.0001). Caregiving for patients treated with surgery (83.86 ± 30.88) was associated with significantly greater scores of Role Emotional compared to those under chemo + radiation therapy (45.03 ± 42.89, P < 0.0001), radiation therapy (46.96 ± 46.75, P = 0.022), and chemotherapy (56.50 ± 42.86, P = 0.014).

No significant relationships were observed between other basic characteristic of the participants and Role Emotional (All, P > 0.05). (Tables 3 and 4)

#### Mental health

There was a significant association between duration of caregiving (F (2, 297) = 44.259, P < 0.0001) and Mental Health, so that, caregiving for patients 6–12 months (70.36 ± 24.17) was related to significantly higher levels of Mental Health compared to 12–23 (68.95 ± 23.67, P < 0.0001) and ≥ 24 (68.17 ± 24.99, P < 0.0001) months.

Similarly, time since diagnosis showed a significant association with Mental Health (F (2, 297) = 43.046, P < 0.0001). Caregiving for patients with 6–12 months since their diagnosis (69.09 ± 18.27) was related to significantly greater levels of Mental Health compared to 12–23 (62.13 ± 18.67, P < 0.0001) and ≥ 24 (44.42 ± 19.43, P < 0.0001) months.

No other significant associations were observed between basic characteristic of the participants and Mental Health (All, P > 0.05). (Tables 3 and 4)

### Predicting factors for quality of life domains

#### Physical functioning

Eight models and seven significant predictors were found in terms of caregiver Physical Functioning including, employment status (Retired), ECOG [4], stage of cancer [3], duration of caregiving (≥ 24 months), ECOG [3], employment status (Governmental employed), type of cancer (Brain).

The first model suggests being retired as a significant predicting factor of Physical Functioning. According to the R^2^ value of this model (R^2^ = 0.05), being retired accounts for 5% of the variation in Physical Functioning, indicating that 95% of the variation in the Physical Functioning cannot be explained by employment status (Retired) alone. The regression coefficient [B = -12.86, 95%CI (-18.78, -6.94), P < 0.001] demonstrated that being retired resulted in 12.86 units lower Physical Functioning than other categories.

In the second model, ECOG [4] was added to the analysis. The R^2^ value of 0.07 associated with this model indicates that the addition of ECOG [4] to the first model accounts for 7% of the variation in caregiver Physical Functioning, which means that 93% of its variation cannot be explained by employment status (Retired) and ECOG [4] alone. Controlling for ECOG [4], the regression coefficient [B = -12.89, 95%CI (-18.74, -7.03), P < 0.001] showed that being retired leads to 12.89 units lower Physical Functioning than other categories. Controlling for employment status (Retired), the regression coefficient [B = -22.07, 95%CI (-38.02, -6.11), P < 0.001] associated with ECOG [4] revealed that caregivers of patients in ECOG 4 category experienced 22.07 units lower levels of Physical Functioning compared to other categories. Table 5 shows all eight regression models thoroughly.

**Table 5:** Predicting factors for caregiver Physical Functioning according to stepwise linear regression models (N = 300)

Independent variables	Model 1	Model 2	Model 3	Model 4	Model 5	Model 6	Model 7	Model 8
**F value**	18.27	13.03	10.71	9.41	8.67	8.16	7.65	8.45
**Corrected R-squared**	0.05	0.07	0.08	0.10	0.11	0.12	0.13	0.13
Coefficients (95% CI)								
Constants	77.10*** (74.61, 79.59)	77.55*** (75.06, 80.03)	75.68*** (72.77, 78.59)	76.95*** (73.85, 80.05)	77.94*** (74.75, 81.13)	76.47*** (73.04, 79.89)	77.41*** (73.88, 80.94)	78.84*** (75.76, 81.91)
(1) Employment status (Retired)	-12.86*** (-18.78, -6.94)	-12.89*** (-18.74, -7.03)	-12.80*** (-18.62, -6.99)	-12.31*** (-18.10, -6.53)	-12.61*** (-18.36, -6.86)	-11.06*** (-16.94, -5.19)	-11.15*** (-16.99, -5.30)	-11.20*** (-17.06, -5.34)
(2) ECOG (4)		-22.07** (-38.02, -6.11)	-20.21* (-36.12, -4.30)	-21.56** (-37.41, -5.72)	-22.50** (-38.26, -6.75)	-22.34** (-37.99, -6.70)	-23.25** (-38.85, -7.66)	-24.68** (-40.22, -9.15)
(3) Stage of cancer (3)			5.80* (1.00, 10.61)	5.59* (0.82, 10.36)	4.78 (-0.009, 9.57)	4.66 (-0.09, 9.42)	3.90 (-0.89, 8.69)	
(4) Duration of caregiving (≥ 24 months)				-6.33* (-11.86, -0.79)	-7.02* (-12.55, -1.49)	-7.14* (-12.63, -1.64)	-7.35** (-12.82, -1.88)	-7.61** (-13.09, -2.14)
(5) ECOG (3)					-13.14* (-24.49, -1.78)	-13.90* (-25.20, -2.60)	-14.80* (-26.08, -3.53)	-16.25** (-27.41, -5.09)
(6) Employment status (Governmental employed)						6.32* (0.75, 11.88)	6.21* (0.67, 11.74)	6.29* (0.74, 11.83)
(7) Type of cancer (Brain)							-9.47* (-18.70, -0.23)	-10.64* (-19.78, -1.49)

*p < 0.05, **p < 0.01, ***p < 0.001

#### Bodily pain

Five models and five significant predictors were secured for caregiver Bodily Pain including, ECOG [4], patient’s Gender (Female), ECOG [3], ECOG [2], and education (bachelor’s degree).

In the first model ECOG [4] was recommended as a significant predictor for caregiver Bodily Pain.

The R^2^ value of this model (R2 = 0.03) signifies that caring for ECOG 4 patients justifies 3% of the variation in Bodily Pain, indicating that 97% of its variation cannot be explained by ECOG [4] alone. The regression coefficient [B = -21.90, 95%CI (-35.32, -8.49), P < 0.001] revealed that caring for patients in ECOG 4 category resulted in 21.90 units lower Bodily Pain than other categories.

In the second model, patient’s gender (Female) was added to the analysis. The R^2^ value of 0.04 associated with this model indicates that the addition of patient’s gender (Female) to the first model accounts for 4% of the variation in caregiver Bodily Pain, which means that 96% of its variation cannot be clarified by ECOG [4] and caring for female patients alone. Controlling for patient’s gender (Female), the regression coefficient [B = -23.85, 95%CI (-37.21, -10.49), P < 0.001] showed that caring for patients in ECOG 4 category results in 23.85 units lower Bodily Pain than other categories. Controlling for ECOG [4], the regression coefficient [B = -5.02, 95%CI (-8.78, -1.26), P < 0.001] associated with patient’s gender (Female) revealed that caregivers of female patients experienced 5.02 units lower levels of Bodily Pain compared to caregivers of male patients. Table 6 shows all five regression models in detail.

**Table 6:** Predicting factors for caregiver Bodily Pain according to stepwise linear regression models (N = 300)

Independent variables	Model 1	Model 2	Model 3	Model 4	Model 5
**F value**	10.33	8.73	7.53	7.10	6.79
**Corrected R-squared**	0.03	0.04	0.06	0.07	0.08
Coefficients (95% CI)					
Constants	76.90*** (75.01, 78.80 )	84.71*** (78.58, 90.85)	85.52*** (79.38, 91.66)	86.80*** (80.62, 92.99)	86.52*** (80.37, 92.67)
(1) ECOG (4)	-21.90*** (-35.32, -8.49)	-23.85*** (-37.21, -10.49)	-24.38*** (-37.66, -11.10)	-25.41*** (-38.62, -12.20)	-27.01*** (-40.21, -13.82)
(2) Patient’s Gender (Female)		-5.02** (-8.78, -1.26)	-5.26** (-9.00, -1.52)	-5.48** (-9.19, -1.76)	-5.71** (-9.41, -2.02)
(3) ECOG (3)			-10.65* (-20.09, -20.09)	-11.62* (-21.03, -2.21)	-12.09* (-21.44, -2.73)
(4) ECOG (2)				-6.32* (-11.64, -1.00)	-7.07** (-12.40, -1.75)
(5) Education (Bachelor’s degree)					6.47* (0.86, 78.80 )

*p < 0.05, **p < 0.01, ***p < 0.001

#### General Health

Time since diagnosis (≥ 24 months), type of treatment (Radiation therapy), time since diagnosis (12–23 months), ECOG [2] and ECOG [3] significantly predicted caregiver General Health.

First model suggested time since diagnosis (≥ 24 months) as a significant predictor for caregiver General Health. The R^2^ value of this model (R2 = 0.24) denotes that caring for patients with ≥ 24 months since diagnosis explains 24% of the variation in General Health, indicating that 76% of its variation cannot be explained by time since diagnosis (≥ 24 months) alone. The regression coefficient [B = -24.72, 95%CI (-29.71, -19.74), P < 0.001] indicated that caring for patients with ≥ 24 months since diagnosis resulted in 24.72 units lower General Health than other categories.

Type of treatment (Radiation therapy) was added to the analysis in the second model. The R^2^ value of 0.25 associated with this model indicates that the addition of Type of treatment (Radiation therapy) to the first model accounts for 25% of the variation in caregiver General Health, which means that 75% of its variation cannot be clarified by time since diagnosis (≥ 24 months) and type of treatment (Radiation therapy) alone. Controlling for type of treatment (Radiation therapy), the regression coefficient [B = -24.71, 95%CI (-29.66, -19.76), P < 0.001] showed that caring for patients with ≥ 24 months since diagnosis leads to 24.71 units lower General Health than other categories. Controlling for time since diagnosis (≥ 24 months), the regression coefficient [B = 9.18, 95%CI (1.19, 17.17), P < 0.05] associated with type of treatment (Radiation therapy) uncovered that caregivers of patients who were under radiation therapy experienced 9.18 units greater levels of General Health compared to other categories. Table 7 shows all five regression models in detail.

**Table 7:** Predicting factors for caregiver General Health according to stepwise linear regression models (N = 300)

Independent variables	Model 1	Model 2	Model 3	Model 4	Model 5
**F value**	95.32	50.87	36.20	28.91	24.30
**Corrected R-squared**	0.24	0.25	0.26	0.27	0.28
Coefficients (95% CI)					
Constants	57.33*** (54.94, 59.72)	56.66*** (54.21, 59.10)	58.31*** (55.50, 61.11)	59.23*** (56.34, 62.12)	59.69*** (56.79, 62.60)
(1) Time since diagnosis (≥ 24 months)	-24.72*** (-29.71, -19.74)	-24.71*** (-29.66, -19.76)	-26.37*** (-31.49, -21.26)	-26.25*** (-31.33, -21.17)	-26.64*** (-31.70, -21.58)
(2) Type of treatment (Radiation therapy)		9.18* (1.19, 17.17)	9.34* (1.40, 17.27)	10.35* (2.43, 18.27)	10.47** (2.59, 18.35)
(3) Time since diagnosis (12–23 months)			-6.29* (-11.63, -0.94)	-6.48* (-11.79, -1.17)	-5.78* (-11.10, -0.46)
(4) ECOG (2)				-7.04* (-12.99, -1.08)	-7.53* (-13.47, -1.59)
(5) ECOG (3)					-11.43* (-22.02, -0.83)

*p < 0.05, **p < 0.01, ***p < 0.001

#### Vitality

Stepwise linear regression analysis of Vitality resulted in five models and five significant predictors. Age of caregiver, stage of cancer [1], type of cancer (Prostate), duration of caregiving (≥ 24 months) and health insurance (Social security) were significant predictors of Vitality.

The first model suggests age of caregiver as a significant predicting factor of Vitality. According to the R^2^ value of this model (R^2^ = 0.18), age of caregiver accounts for 18% of the variation in Vitality, indicating that 82% of the variation in the Vitality cannot be explained by age of caregiver alone. The regression coefficient [B = -0.69, 95%CI (-18.78, -6.94), P < 0.001] demonstrated that caregivers with older age experience 0.69 units lower Vitality than younger ones.

In the second model, stage of cancer [1] was added to the analysis. The R^2^ value of 0.21 associated with this model showed that the addition of stage of cancer [1] to the first model accounts for 21% of the variation in caregiver Vitality, which means that 79% of its variation cannot be justified by age of caregiver and stage of cancer [1] alone. Controlling for stage of cancer [1], the regression coefficient [B = -0.69, 95%CI (-0.85, -0.53), P < 0.001] showed that caregivers with older age resulted in 0.69 units lower Vitality compared with younger ones. Controlling for age of caregiver, the regression coefficient [B = 13.57, 95%CI (6.77, 20.38), P < 0.001] associated with stage of cancer [1] revealed that caregivers of patients with stage 1 cancer experienced 13.57 units greater levels of Vitality compared to caregivers of other categories. Table 8 shows all five regression models in detail.

**Table 8:** Predicting factors for caregiver Vitality according to stepwise linear regression models (N = 300)

Independent variables	Model 1	Model 2	Model 3	Model 4	Model 5
**F value**	66.46	42.55	35.06	30.97	26.71
**Corrected R-squared**	0.18	0.21	0.25	0.28	0.30
Coefficients (95% CI)					
Constants	75.67*** (68.56, 82.77)	74.44*** (67.47, 81.40)	72.66*** (65.80, 79.51)	74.53*** (67.75, 81.31)	74.92*** (68.20, 81.64)
(1) Age of caregiver	-0.69*** (-0.85, -0.52)	-0.69*** (-0.85, -0.53)	-0.68*** (-0.84, -0.52)	-0.68*** (-0.84, -0.53)	-0.64*** (-0.80, -0.49)
(2) Stage of cancer (1)		13.57*** (6.77, 20.38)	14.71*** (8.04, 21.37)	14.68*** (8.16, 21.21)	13.81*** (7.32, 20.30)
(3) Type of cancer (Prostate)			12.76*** (6.45, 19.06)	12.51*** (6.33, 18.68)	11.88*** (5.75, 18.00)
(4) Duration of caregiving (≥ 24 months)				-9.28*** (-14.15, -4.40)	-9.36*** (-14.19, -4.54)
(5) Health insurance (Social security)					-5.71** (-9.93, -1.49)

**p < 0.01, ***p < 0.001

#### Social functioning

Care setting (Outpatient), ECOG [3], ECOG [4], ECOG [4], ECOG [2] and Type of cancer (Prostate) significantly predicted caregiver Social Functioning.

The first model suggests outpatient care setting as a significant predicting factor of Social Functioning. According to the R^2^ value of this model (R^2^ = 0.05), Care setting (Outpatient) accounts for 5% of the variation in Social Functioning, indicating that 95% of the variation in the Social Functioning cannot be explained by care setting alone. The regression coefficient [B = 12.02, 95%CI (6.69, 17.35), P < 0.001] demonstrated that caregivers in outpatient care setting experience 12.02 units higher Social Functioning than caregivers in inpatient care setting.

In the second model, ECOG [3] was added to the analysis. The R^2^ value of 0.07 associated with this model showed that the addition of ECOG [3] to the first model accounts for 7% of the variation in caregiver Social Functioning, which means that 93% of its variation cannot be justified by Care setting (Outpatient) and ECOG [3] alone. Controlling for ECOG [3], the regression coefficient [B = 11.30, 95%CI (6.00, 16.61), P < 0.001] showed that caregivers in outpatient care setting experienced 11.30 units higher levels of Social Functioning in comparison to caregivers in inpatient care setting. Controlling for Care setting (Outpatient), the regression coefficient [B = -18.47, 95%CI (-32.00, -4.94), P < 0.001] associated with ECOG [3] revealed that caregivers of patients in ECOG 3 experienced 18.47 units lower levels of Social Functioning compared to caregivers of other categories. Table 9 shows all five regression models in detail.

**Table 9:** Predicting factors for caregiver Social Functioning according to stepwise linear regression models (N = 300)

Independent variables	Model 1	Model 2	Model 3	Model 4	Model 5	Model 6
**F value**	19.70	13.66	11.40	10.00	9.31	9.05
**Corrected R-squared**	0.05	0.07	0.09	0.10	0.12	0.13
Coefficients (95% CI)						
Constants	51.69*** (43.29, 60.08)	53.50*** (45.08, 61.91)	54.99*** (46.57, 63.41)	56.85*** (48.34, 65.36)	57.60*** (49.13, 66.06)	58.82*** (50.39, 67.25)
(1) Care setting (Outpatient)	12.02*** (6.69, 17.35)	11.30*** (6.00, 16.61)	10.65*** (5.37, 15.93)	10.72*** (5.48, 15.96)	11.30*** (6.08, 16.52)	9.85*** (4.57, 15.14)
(2) ECOG (3)		-18.47** (-32.00, -4.94)	-19.13** (-32.55, -5.72)	-19.97** (-33.31, -6.63)	-21.31** (-34.59, -8.03)	-23.51** (-36.76, -10.25)
(3) ECOG (4)			-24.08* (-42.85, -5.31)	-23.79* (-42.43, -5.16)	-24.95** (-43.45, -6.44)	-28.20** (-46.69, -9.70)
(4) ECOG (4)				-6.71* (-12.44, -0.98)	-7.52* (-13.24, -1.80)	-7.49* (-13.15, -1.82)
(5) ECOG (2)					-9.29** (-16.83, -1.75)	-10.56** (-18.08, -3.03)
(6) Type of cancer (Prostate)						11.11* (2.73, 19.49)

*p < 0.05, **p < 0.01, ***p < 0.001

#### Role emotional

Relationship to the patient (Offspring), stage of cancer [1], type of cancer (Bladder), relationship to the patient (Siblings), relationship to the patient (Others), stage of cancer [3], education (Primary School), care setting (Outpatient), type of treatment (Chemo Radiotherapy) significantly predicted caregiver Role Emotional.

The first model suggests Relationship to the patient (Offspring) as a significant predicting factor of Role Emotional. According to the R^2^ value of this model (R^2^ = 0.11), relationship to the patient (Offspring) accounts for 11% of the variation in Role Emotional, indicating that 89% of its variation cannot be explained by relationship to the patient (Offspring) alone. The regression coefficient [B = 29.96, 95%CI (20.74, 39.19), P < 0.001] demonstrated that being offspring to the patients resulted in 29.96 units higher levels of Role Emotional than other categories.

In the second model, stage of cancer [1] was added to the analysis. The R^2^ value of 0.16 associated with this model showed that the addition of stage of cancer [1] to the first model accounts for 16% of the variation in caregiver Role Emotional, which means that 84% of its variation cannot be explained by relationship to the patient (Offspring) and stage of cancer [1] alone.

Controlling for Stage of cancer [1], the regression coefficient [B = 29.45, 95%CI (20.47, 38.44), P < 0.001] showed that offspring of the patients experienced 29.45 units higher levels of Role Emotional in comparison to caregivers in other categories.

Controlling for relationship to the patient (Offspring), the regression coefficient [B = 31.78, 95%CI (16.81, 46.75), P < 0.001] associated with stage of cancer [1] revealed that caregivers of patients with stage 1 cancer experienced 31.78 units higher levels of Role Emotional compared to caregivers of other categories. Table 10 shows all five regression models in detail.

**Table 10:** Predicting factors for caregiver Role Emotional according to stepwise linear regression models (N = 300)

Independent variables	Model 1	Model 2	Model 3	Model 4	Model 5	Model 6	Model 7	Model 8	Model 9
**F value**	40.89	30.30	26.69	25.41	26.87	25.22	23.52	21.63	19.95
**Corrected R-squared**	0.11	0.16	0.20	0.24	0.30	0.32	0.34	0.35	0.36
Coefficients (95% CI)									
Constants	42.09*** (35.61, 48.57)	39.16*** (32.71, 45.62)	40.57*** (34.24, 46.90)	35.85*** (29.29, 42.41)	28.61*** (21.68, 35.55)	34.50*** (26.91, 42.08)	37.13*** (29.45, 44.80)	22.22*** (7.81, 36.64)	23.458*** (9.08, 37.83)
(1) Relationship to the patient (Offspring)	29.96*** (20.74, 39.19)	29.45*** (20.47, 38.44)	31.85*** (23.01, 40.68)	36.66*** (27.76, 45.56)	43.75*** (34.73, 52.76)	43.17*** (34.31, 52.03)	42.29*** (33.53, 51.04)	41.50*** (32.79, 50.22)	41.54*** (32.88, 50.20)
(2) Stage of cancer (1)		31.78*** (16.81, 46.75)	31.41*** (16.82, 46.01)	31.06*** (16.84, 45.27)	32.69*** (19.00, 46.38)	27.26*** (13.47, 41.06)	28.47*** (14.84, 42.10)	23.81*** (9.76, 37.86)	22.15*** (8.10, 36.21)
(3) Type of cancer (Bladder)			-34.78*** (-51.72, -17.84)	-35.26*** (-51.76, -18.77)	-35.52*** (-51.39, -19.64)	-36.89*** (-52.49, -21.28)	-35.13*** (-50.56, -19.69)	-31.03*** (-46.71, -15.35)	-29.91*** (-45.54, -14.29)
(4) Relationship to the patient (Siblings)				38.16*** (20.05, 56.27)	45.24*** (27.59, 62.89)	44.14* (26.80, 61.48)	44.81** (27.69, 61.92)	45.90*** (28.90, 62.89)	47.33*** (30.38, 64.28)
(5) Relationship to the patient (Others)					41.06*** (24.75, 57.36)	41.62*** (25.61, 57.64)	40.16*** (24.33, 55.98)	40.84*** (25.14, 56.55)	39.52*** (23.85, 55.18)
(6) Stage of cancer (3)						-15.72** (-24.66, -6.78)	-15.16** (-23.99, -6.34)	-15.39** (-24.15, -6.64)	-13.51** (-22.40, -4.63)
(7) Education (Primary School)							-17.38** (-28.71, -6.06	-16.97** (-28.21, -5.73)	-17.04** (-28.21, -5.87)
(8) Care setting (Outpatient)								10.27* (1.83, 18.71)	10.78* (2.38, 19.18)
(9) Type of treatment (Chemo Radiotherapy)									-10.11* (-19.55, -0.66)

*p < 0.05, **p < 0.01, ***p < 0.001

#### Mental health

Time since diagnosis (≥ 24 months), duration of caregiving (12–23 months), stage of cancer [1], type of cancer (Prostate), and duration of caregiving (≥ 24 months) were significant predictors of Mental Health.

The first model suggests time since diagnosis (≥ 24 months) as a significant predicting factor of Mental Health. According to the R^2^ value of this model (R^2^ = 0.20), caring for patients with ≥ 24 months since diagnosis accounts for 20% of the variation in Mental Health, indicating that 80% of the variation in the Mental Health cannot be explained by time since diagnosis (≥ 24 months) alone. The regression coefficient [B = -22.83, 95%CI (-27.90, -17.75), P < 0.001] demonstrated that caring for patients with ≥ 24 months since diagnosis was associated with 22.83 units lower levels of Mental Health than other categories.

In the second model, duration of caregiving (12–23 months) was added to the analysis. The R^2^ value of 0.22 associated with this model denotes that the addition of duration of caregiving (12–23 months) to the first model accounts for 22% of the variation in caregiver Mental Health, which means that 78% of its variation cannot be clarified by time since diagnosis (≥ 24 months), duration of caregiving (12–23 months) alone. Controlling for duration of caregiving (12–23 months), the regression coefficient [B = -23.53, 95%CI (-28.58, -18.48), P < 0.001] showed that caring for patients with ≥ 24 months since diagnosis was associated with 23.53 units lower levels of Mental Health compared to other categories. Controlling for time since diagnosis (≥ 24 months), the regression coefficient [B = -7.34, 95%CI (-12.76, -1.93), P < 0.01] associated with duration of caregiving (12–23 months) revealed that caregiving for 12–23 months resulted in 7.34 units lower levels of Mental Health compared to other categories. Table 11 shows all five regression models in detail.

**Table 11:** Predicting factors for caregiver Mental Health according to stepwise linear regression models (N = 300)

Independent variables	Model 1	Model 2	Model 3	Model 4	Model 5	Model 6
**F value**	78.44	43.59	30.80	24.58	20.95	25.55
**Corrected R-squared**	0.20	0.22	0.23	0.24	0.25	0.24
Coefficients (95% CI)						
Constants	67.25*** (64.81, 69.68)	68.81*** (66.14, 71.47)	68.01*** (65.25, 70.77)	67.03*** (64.15, 69.91)	67.45*** (64.56, 70.33)	67.32*** (64.44, 70.21)
(1) Time since diagnosis (≥ 24 months)	-22.83*** (-27.90, -17.75)	-23.53*** (-28.58, -18.48)	-23.40*** (-28.42, -18.37)	-23.12*** (-28.12, -18.13)	-9.55 (-22.41, 3.29)	
(2) Duration of caregiving (12–23 months)		-7.34** (-12.76, -1.93)	-7.22** (-12.60, -1.83)	-7.10** (-12.46, -1.75)	-9.50** (-15.22, -3.79)	-10.78*** (-16.25, -5.32)
(3) Stage of cancer (1)			7.36* (0.35, 14.37)	8.02* (1.03, 15.02)	8.45* (1.50, 15.40)	8.77* (1.81, 15.72)
(4) Type of cancer (Prostate)				7.32* (0.70, 13.94)	7.72* (1.13, 14.30)	8.05* (1.47, 14.63)
(5) Duration of caregiving (≥ 24 months)					-15.84* (-29.69, -2.00)	-25.34* (-30.70, -19.98)

*p < 0.05, **p < 0.01, ***p < 0.001

## Discussion

This study resulted in three considerable findings: (1) Low levels of the QoL domains among caregivers of cancer patients, namely role emotional, general health and vitality with the lowest mean values among others (2) Significant differences of QoL domains between categories of some basic and clinical characteristics of patients and their caregivers (3) Some significant predicting factors for QoL domains.

We revealed that in terms of the gender of the participant, it was a significant predictor in none of the eight subscales, conversely, the gender of the patient showed a significant effect, so that, caregivers of female patients experienced about 5 units lower levels of bodily pain in comparison to males. No differences between men and women QoL levels have been previously reported [3]. Even so, lower scores of QoL among female caregivers have been reported in several studies [18–22]. Almutairi et al. has shown that in the subscales of role emotional as well as energy/fatigue, emotional well-being, pain, and general health, female caregivers reported significantly lower functioning scores compared to males [20].

It is claimed that the high responsibility of women in the society tends to cause such low QoL in women. Given the fact that in some traditional societies, where women play numerous roles in household chores and child-rearing tasks, caring for patients has been added to the potential responsibilities of women and can cause a lower perceived QoL [19]. Evidence shows that in Iran, contrary to the promising trend of women’s health during the last three decades, there are still significant differences between women and men in terms of physical, mental and social health [23]. Thus, potentially, there are health disparities between men and women in Iran, and the low QoL among female caregivers may also be partly derived from this background difference.

Lim et al. has confirmed that being a male family caregiver of cancer patients is significantly associated with lower levels of QoL [12].

With regard to the age of caregivers, we demonstrated that elderly caregivers may develop 0.69 units less vitality than younger ones. In line with our findings, in a Brazilian population of cancer caregivers, caregivers who aged ≥ 60 years experienced significantly lower levels of QoL. In the aforementioned study the mean scores of SF-36 domains were remarkably low as role emotional (14.7 ± 31.9), role physical (26.8 ± 37.5) and vitality (35.9 ± 27.9) showed the lowest mean scores, respectively [24]. Another study has proved that older cancer caregivers have significantly lower scores pertaining to physical functioning and social functioning [20].

The findings of our study also suggests a number of predictors including: duration of caregiving as well as ECOG, type and stage of cancer, type of treatment, education status, care setting, relationship to the patient and type of health insurance. Our results corroborates the existing evidence [8, 12, 22]. In a study by Rha et al. the results have indicated that caregiving burden was a significant predictor of the QoL and caring for patients who had functional impairment was associated with higher burden. Caregivers of inpatients along with those who had lower educational level also experienced lower QoL [8].

Abdullah et al. [22]. In a study to investigate the QoL of GI cancer patients and their family caregivers, intriguing results have been achieved. They confirmed that there was no significant relationship between the demographic variables of the caregivers and the level of their QoL, while there was a significant association between ethnicity, time since diagnosis, primary cancer site, and surgery of the patients and their QoL, so that, higher levels of QoL was related to longer cancer duration, having lower gastrointestinal (GI) cancer, Chinese ethnicity, and having surgery. There was also a significant correlation between all SF-12 domains between patients and their family caregivers. According to our findings, caregivers of patients who were receiving chemoradiotherapy experienced about 10 points lower levels of QoL (role emotional) compared to others.

We showed that duration of caregiving (≥ 24 months) was a significant predictor for vitality, mental health and physical functioning domains. This may be a result of increased unmet needs and perceived distress among caregivers with longer durations of caregiving and time since cancer diagnosis. As evidenced by Yang et al., among 237 family caregivers of cancer patients, compared to early treatment phase (< 6 months), there was a significant link between unmet personal care needs of the participants and higher overall distress and stress in the intermediate treatment phase (6–9 months). Moreover, in the chronic treatment phase (> 9 months), higher unmet personal care needs were related to significantly greater levels of distress, anxiety and stress [25].

We found health insurance and employment status as significant determining factors for vitality and physical functioning, respectively. Caregivers of patients with social security insurance experienced about 5 point less QoL (vitality). Caregivers who were governmental employed had about 6 points better level of physical functioning, retired caregivers experienced about 11 points less physical functioning compared to others, though. These factors can be seen from the perspective of social support necessary for caregivers. A literature review has indicated that caregivers who are provided with poor social support, experience lower QoL. In other words, when working conditions worsen for these individuals, and the time allocated for developing daily activities or spending time for themselves decreases, it can cause a decline in the quality of life indicators [26].

Another significant predictor secured was education status. Caregivers with primary school level of education showed about 17 points less role emotional score, conversely, caregivers with bachelor’s degree had about 6 points higher levels of bodily pain. It has already been shown that a high level of education might be related to a significantly better quality of life [19]. On one hand, such findings may be on account of the fact that having a high level of education leads to better communication skills and a better comprehension of stress management and coping strategies. On the other hand, caregivers with low education may not be able to competently address the therapeutic needs of their patients and this would negatively affect their QoL [19].

Stage of cancer and ECOG were among the frequently observed predictors.

Rosa et al. did not find a significant association between any of the SF-36 domains with ECOG and stage of the cancer [24]. However, Hsu et al. reported that caregivers of patients with poorer performance status were more likely to experience lower levels of QoL [27]. One reason for this might be the imminence of a loved-one’s death that sounds to be excessively depressing and such a huge psychological burden on the whole family including the caregivers of the patients, leading to a decrease in QoL levels.

What emphasizes more on the importance of examining the QoL not as a whole but by considering the different domains of that, is the findings of the current study and also the existing evidence [3, 12, 20, 24, 28, 29], signifying the fact that the predictive variables of each of the domains of QoL may be completely distinct from the others.

It has been inferred that palliative care for hospitalized patients undergoing hematopoietic stem cell transplantation, produced significant improvement in administrative and financial domains of QoL whereas, nurse home visits and telephone sessions significantly affected social and emotional domains, and not functional ones [28].

Another factor that is particularly important and seems to have been neglected in the existing literature, is the sociocultural and religious background of individuals, which could still cause Inequalities in the perceived QoL domains in different populations even with homogeneous demographic and clinical influencing factors. The fact that it is not only about subjective factors influencing the low QoL, but also the caregiver’s objective characteristics such as the resilience, adaptation and coping strategies and how effectively they could resist the crises are partly to blame for such outcomes [12]. It has been reported that family caregivers of cancer patients in Singapore and Asia may experience lower QoL compared to their Western equivalents. In Asia, caregivers residing in countries like Singapore, Turkey, and Taiwan have been shown to experience better QoL compared to ones in Iran and South Korea [12].

Finally, it is crucial to acknowledge that when assessing the mental health and quality of life of patients and caregivers, it is not only about focusing on the individuals. The caregiver-patient dyads should also be taken into consideration as a strong bond between them can play a significant role in preventing psychological distress, improving quality of life, and increasing relationship satisfaction [4].

### Limitations

This study faced a number of limitations. First, the cross-sectional nature of the study design does not allow for the establishment of causal relationships between variables. Second, convenience sampling may result in a non-representative sample, as it may not include caregivers with varied backgrounds and experiences. This can limit the generalizability of the findings to a broader population of cancer caregivers. Third, self-Report bias is another limitation as the use of self-reported QoL measures, such as the SF-36, can introduce social desirability bias, where respondents may furnish responses they think are socially expected rather than genuinely reflecting their actual experiences. Lastly, relying solely on one tool to measure QoL may not capture the full complexity of factors affecting QoL. Other important factors, such as psychological distress, social support, or specific caregiving-related challenges, might be overlooked. We recommend conducting longitudinal studies and clinical trials in a more diverse and representative sampling method, incorporating multiple measurement tools, and conducting longitudinal research to provide a more comprehensive understanding of QoL among cancer caregivers.

## Conclusion

In summary, we revealed that caregivers of cancer patients experienced low levels of QoL. There were various significant predicting factors for QoL domains. Such findings imply the unmet needs of cancer caregivers and probably the neglected importance of their QoL for clinicians and healthcare policy makers.

Acknowledging the factors affecting the QoL among this population can be a crucial step on the road to adopt effective interventional and preventive measures. By recognizing the factors that predict low QoL in caregivers, healthcare teams can proactively identify caregivers at high risk and offer additional support and resources. Healthcare providers can develop targeted support programs for family caregivers to address their specific needs. These programs could focus on improving caregivers’ QoL by offering counseling, educational services, and facilitating access to essential resources.

The study may serve as a catalyst for additional research, seeking a more in-depth understanding of the distinct requirements and experiences of family caregivers across diverse populations and various contexts. This can be achieved through the utilization of more objective assessment tools rather than solely relying on questionnaire-based studies. Furthermore, further exploration of the efficacy of interventions designed to improve the QoL for family caregivers should be pursued.

## Data Availability

The datasets used and analyzed during the current study are available from the corresponding author on reasonable request.

## Abbreviations

QoL: Quality of Life
SF-36: Medical Outcomes General Health Survey Short Form 36
ECOG: Eastern Cooperative Oncology Group
SD: Standard Deviation
IQR: InterQuartile Range
ANOVA: Analysis of Variance
HSD: Honestly Significant Difference
HSD: High School Diploma
BS: Bachelor’s Degree
MS: Master’s Degree
GI: GastroIntestinal
